# Bioinformatic analysis and functional predictions of selected regeneration-associated transcripts expressed by zebrafish microglia

**DOI:** 10.1186/s12864-020-07273-8

**Published:** 2020-12-07

**Authors:** Ousseini Issaka Salia, Diana M. Mitchell

**Affiliations:** 1 Department of Biological Sciences, University of Idaho, Moscow, ID, USA; 2 Institute for Modeling Collaboration and Innovation (IMCI), University of Idaho, Moscow, ID, USA; 3 Present affiliation: Kellog Biological Station and Department of Plant Biology, Michigan State University, 3700 East Gull Lake Drive, Hickory Corners, MI 49060 USA

**Keywords:** Zebrafish, Retina, Microglia, RNAseq, Regeneration, Transcripts, Bioinformatic analysis, Functional predictions

## Abstract

**Background:**

Unlike mammals, zebrafish have a remarkable capacity to regenerate a variety of tissues, including central nervous system tissue. The function of macrophages in tissue regeneration is of great interest, as macrophages respond and participate in the landscape of events that occur following tissue injury in all vertebrate species examined. Understanding macrophage populations in regenerating tissue (such as in zebrafish) may inform strategies that aim to regenerate tissue in humans. We recently published an RNA-seq experiment that identified genes enriched in microglia/macrophages in regenerating zebrafish retinas. Interestingly, a small number of transcripts differentially expressed by retinal microglia/macrophages during retinal regeneration did not have predicted orthologs in human or mouse. We reasoned that at least some of these genes could be functionally important for tissue regeneration, but most of these genes have not been studied experimentally and their functions are largely unknown. To reveal their possible functions, we performed a variety of bioinformatic analyses aimed at identifying the presence of functional protein domains as well as orthologous relationships to other species.

**Results:**

Our analyses identified putative functional domains in predicted proteins for a number of selected genes. For example, we confidently predict kinase function for one gene, cytokine/chemokine function for another, and carbohydrate enzymatic function for a third. Predicted orthologs were identified for some, but not all, genes in species with described regenerative capacity, and functional domains were consistent with identified orthologs. Comparison to other published gene expression datasets suggest that at least some of these genes could be important in regenerative responses in zebrafish and not necessarily in response to microbial infection.

**Conclusions:**

This work reveals previously undescribed putative function of several genes implicated in regulating tissue regeneration. This will inform future work to experimentally determine the function of these genes in vivo, and how these genes may be involved in microglia/macrophage roles in tissue regeneration.

**Supplementary Information:**

The online version contains supplementary material available at 10.1186/s12864-020-07273-8.

## Background

Tissue regeneration allows restoration of the function of damaged tissues and organs. Mammals have the ability to regenerate a limited number of tissues and organs like skin [1, 2], skeletal muscle [3, 4] and liver [5, 6]. Unfortunately, injuries or disease of the central nervous system (CNS) resulting in neuronal loss cannot regenerate neurons in mammals [7–12]. In contrast, zebrafish (*Danio rerio*) have the ability to regenerate numerous different tissues, including tissue in the central nervous system [10, 12–19]. For example, zebrafish can regenerate damaged retinal neurons, which restores visual function [20]. In all species examined, macrophage populations appear to be crucial to tissue regeneration [21–30], though in the mammalian CNS they appear to instead engage in pathological functions [31–35].

In vertebrates, the retina lies at the back of the eye and is a stereotypically organized part of the CNS that is composed of neural and glial cell types that are laminated into 3 distinct nuclear layers. Evidence strongly indicates that Müller glia are the source of regenerated retinal neurons in zebrafish [12, 36–42]. In both zebrafish and mammals, resident microglia respond to retinal injury and degeneration. This may lead to immune-Müller glia crosstalk that may shape Müller glia reaction to retinal injury [43–45]. The zebrafish is a relatively new, and powerful, vertebrate model in microglial biology [10, 30, 46–51]. In particular, microglia and macrophage functions in the regeneration of CNS tissue, such as in the zebrafish retina, is just beginning to be explored.

Our recent work has used the zebrafish towards an understanding of microglia and macrophage responses to acute, widespread retinal lesion in zebrafish [30, 51]. In particular, our transcriptome analysis [30] has provided a rich dataset to facilitate an understanding of gene expression in microglia/macrophages in a context of successful CNS regeneration. In order to translate our transcriptome findings in zebrafish [30] to mammals, we examined predicted orthology of differentially expressed genes (DEGs) enriched in zebrafish microglia/macrophages during retinal regeneration. We found that nearly all of the genes examined had predicted orthologs in mouse and human. However, several of these genes did not. Further, the putative function of these genes is largely unknown. As these “non-orthologous” genes comprise a portion of the microglia/macrophage regeneration-associated transcriptome [30], a better understanding of their predicted gene products will facilitate a greater understanding of the similarities and differences in fish and mammalian response to retinal injury. We reason that these genes could play functional importance in determining the outcome of tissue regeneration in zebrafish, and so functional predictions for these genes is necessary to inform future experimental work. This knowledge will also help us better understand evolutionary relationships between mammalian and teleost immunity.

For twelve selected genes without clear human or mouse orthologues, we performed a variety of bioinformatic analyses aimed to identify functional protein domains. These analyses included identification of protein domains and Gene Ontology (GO) analysis, sequence similarity comparisons, and predicted protein structure. In addition, we used synteny analysis which failed to find evidence of orthologous genes in human and mouse genomes. However, sequence similarity comparisons to find similar genes in other vertebrate species with well described regenerative capacity (Axolotl, *Xenopus*, Salamander) indicated possible orthologs for several of the genes of interest. We also examined several other published gene expression datasets to determine if these genes showed informative expression patterns in other contexts of tissue regeneration, or if these genes might also be differentially expressed in macrophages responding to microbial infection. The work presented here is informative for several zebrafish genes of previously unknown function, providing a foundation for future experimental work to test gene function in vivo. In addition, only one of these twelve genes was previously described to be differentially expressed in macrophages responding to microbial infection, suggesting that these genes indeed have importance to tissue regeneration and not only macrophage responses in general. These results have provided further insight into the transcriptome of zebrafish macrophages in the context of tissue regeneration.

## Results

### Selection of genes expressed in zebrafish microglia/macrophages for further bioinformatics analyses

We previously described a set of 970 genes enriched in in *mpeg1*+ cells (representing microglia and macrophage populations) compared to other retinal cell types in regenerating zebrafish retinas [30]. Of these genes, 409 of them comprised a list that we considered to be “regeneration-associated” transcripts. These particular 409 transcripts were considered to be “regeneration associated” because they were enriched in microglia/macrophages isolated from regenerating retinal tissue, but were not found to be enriched in resting/steady-state zebrafish brain microglia in another published study [30, 52]. Each gene in this list of 409 “regeneration-associated” transcripts was examined for predicted orthology in mouse and human species using the DRSC integrative ortholog prediction tool. Most genes returned predicted orthologues in mouse and/or human (Supplemental File 1). However, twelve (12) of these genes did not show predicted orthology to human or mouse genes with this analysis and were therefore selected for further bioinformatic analysis (Table 1, denoted P1-P12 throughout the manuscript). We reasoned that these twelve transcripts could be part of a transcriptional program executed in microglia/macrophages during CNS regeneration, and therefore could be important in understanding similarities and differences in mammalian vs. zebrafish outcomes following tissue damage.

**Table 1:** Transcripts enriched in zebrafish microglia/macrophages during retinal regeneration, without readily predicted human or mouse orthologs

Gene ID^a^	Mod Log2FC^b^	Zebrafish Symbol^c^	ZFIN ID	Ensembl ID	Chromosome	Gene length	Protein length
P1	6.03	si:dkey-181f22.4	ZDB-GENE-160728-126	ENSDARG00000105643	7	9695 bp	513 aa
P2	5.17	si:ch73-112 l6.1	ZDB-GENE-091204-14	ENSDARG00000093126	21	17,924 bp	1025 aa
P3	2.92	zgc:174863	ZDB-GENE-080204-87	ENSDARG00000099476	6	7668 bp	290 aa
P4	2.14	si:dkey-56 m19.5	ZDB-GENE-030131-226	ENSDARG00000068432	7	4453 bp	526 aa
P5	7.91	si:ch211-105j21.9	ZDB-GENE-131127-499	ENSDARG00000097845	6	2369 pb	294 aa
P6	4.47	si:ch73-248e21.7	ZDB-GENE-120215-231	ENSDARG00000096331	3	3403 bp	480 aa
P7	3.56	si:ch211-191j22.3	ZDB-GENE-030131-4242	ENSDARG00000095459	21	2682 bp	99 aa
P8	7.87	si:ch73-256j6.2^†^	ZDB-GENE-070705-223^†^	ENSDARG00000071653	22	7566 bp	210 aa
P9	7.74	urp1	ZDB-GENE-100922-138	ENSDARG00000093493	14	2696 bp	154 aa
P10	5.32	xcl32a.1	ZDB-GENE-070912-31	ENSDARG00000093906	2	1199 bp	126 aa
P11	6.06	si:ch211-287n14.3	ZDB-GENE-131120-146	ENSDARG00000093650	18	165,070 bp	1809 aa
P12	2.03	pho	ZDB-GENE-030131-5935	ENSDARG00000035133	5	16,478 bp	2798 aa

^a^Gene ID: P1 to P12 correspond to the symbol used for each predicted protein subjected to bioinformatics analysis

^b^Mod Log2FC = Moderated Log2(Fold-Change), which is the log-ratio of the transcript’s expression values between microglia/macrophages and other retinal cells, corrected for lowly expressed transcripts, as determined in [30]

^c^Zebrafish Symbol corresponds to the symbol attributed to each gene by the ZFIN Zebrafish Nomenclature Conventions (https://wiki.zfin.org, [53] and Ensembl ID the symbol attributed by Ensembl (https://www.ensembl.org/, [54]. The prefix “Zgc:” indicates that this gene is represented by cDNAs generated by the ZGC project (https://wiki.zfin.org). The prefix *“si”* Sanger institute and indicates that this institution identified the gene. *aa* amino acid

^†^Previously reported as “NA” in [30] with the same Esembl ID; has been updated here to current zebrafish symbol and ZFIN ID

### Summary of results from bioinformatic analyses

**Fig. 1:**
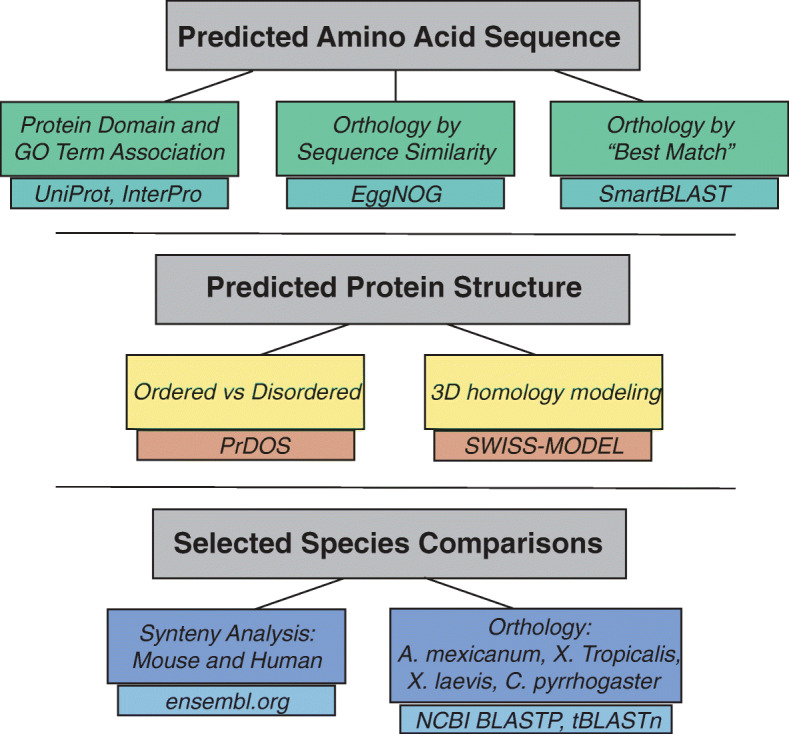
Overview of Bioinformatic Analysis for Functional Predictions. The diagram shows an overview of the bioinformatic analyses performed in order to make functional predictions about the genes of interest based on (**a**) the predicted amino acid sequence, **b** predicted protein structure, and (**c**) genomic comparisons with selected species. The bioinformatic tool used for each type of analysis is indicated. Multiple approaches were used in order to obtain informational results for each gene of interest and to increase confidence in the overall predictions

**Fig. 2:**
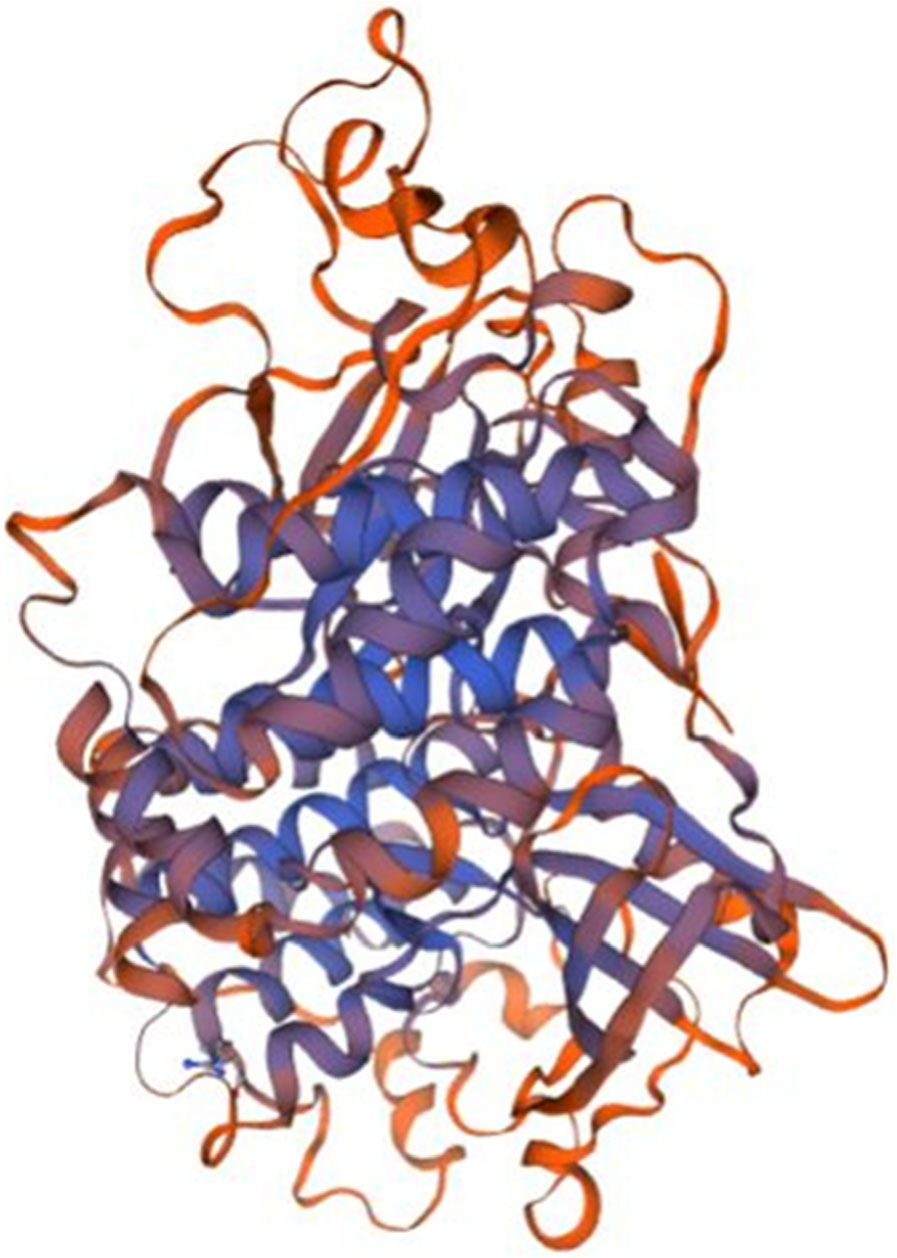
Homology model of P1 putative kinase domain. The kinase domain of Receptor-interacting serine/threonine-protein kinase 2 (RIPK2, 6fu5.1.B in the rcsb protein database) is the template used for the homology modelling of P1. The X-RAY diffraction 3.26 Å was used to determine the experimental structure of 6fu5.1 [60]. The blue color show regions of the model where P1 was well-modeled and orange regions where P1 was poorly modeled. The well-modeled regions (blue) are regions where P1 is likely to be similar to the experimental 3D structure of the template. The homology model pertains to the putative kinase domain of P1 and starts from P1 residue N°3 (GLN, Glutamine) and ends with the residue N° 284 (LYS, Lysine)

**Fig. 3:**
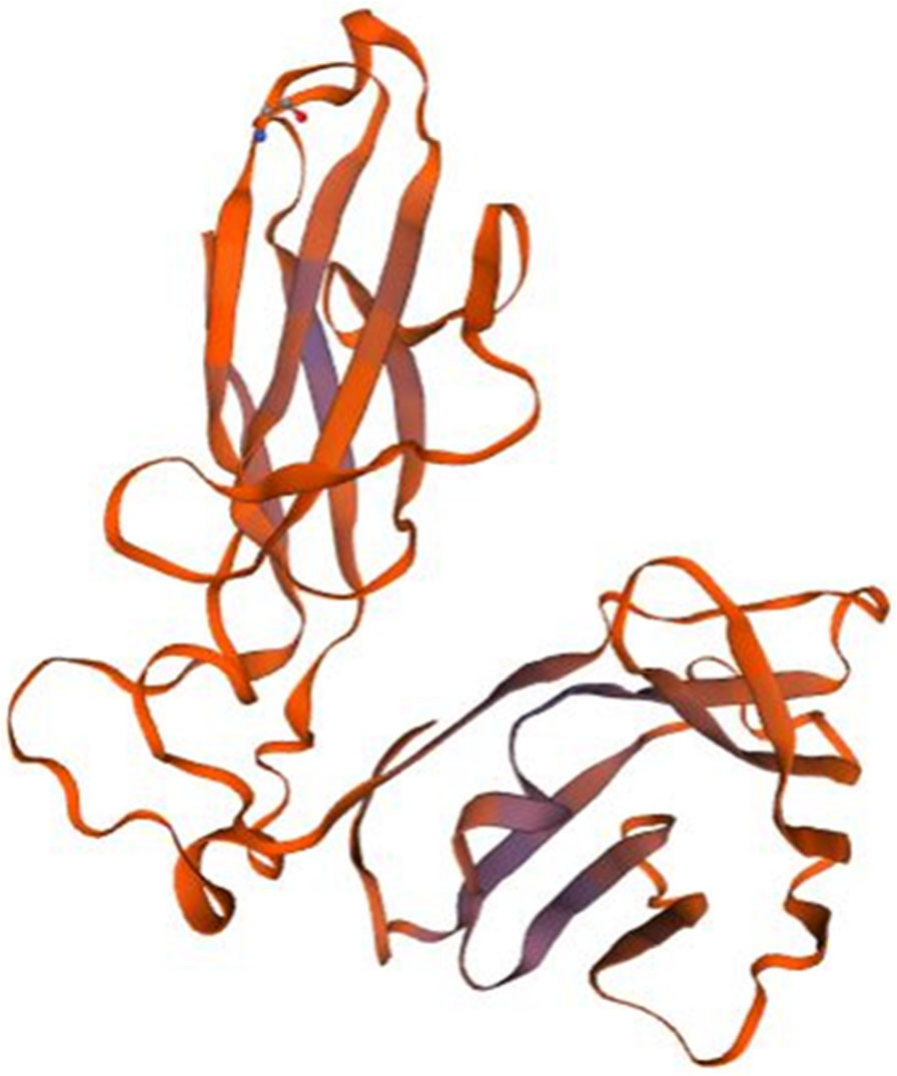
Homology model of P3. T cell receptor beta chain (3of6.1.A in the rcsb protein database) is the template used for the homology modelling of P3. The homology model starts from the P3 residue N°32 (THR, Threonine) and ends with the residue N° 245 (THR, Threonine). The X-RAY diffraction 2.80 Å was used to determine the experimental structure of 3of6.1.A [61]. The blue color show regions of the model in which P3 was well-modeled by the template, and orange regions where P3 was poorly modeled. The blue regions correspond to the T cell receptor beta chain immunoglobulin domains

**Fig. 4:**
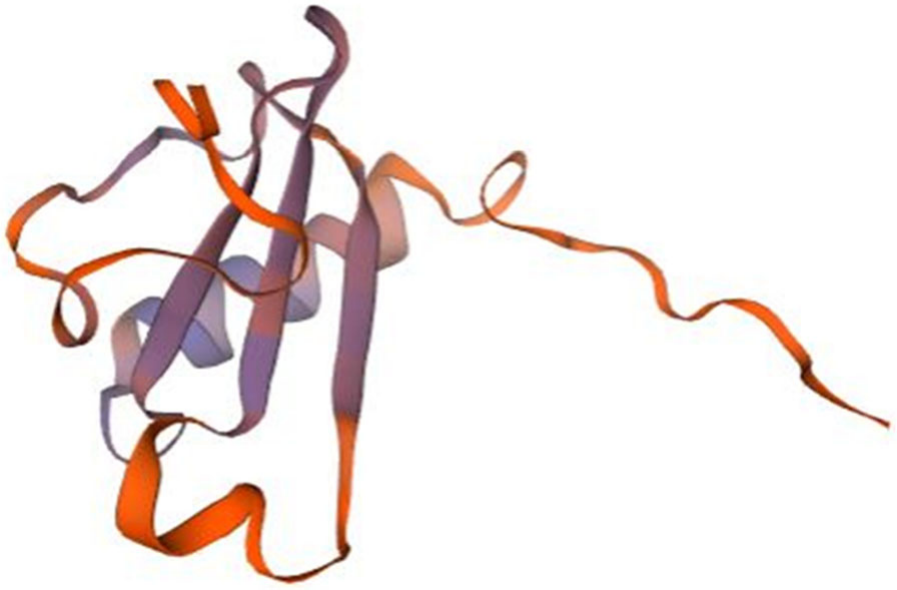
Homology model of P10 chemokine interleukin-8-like domain. Lymphotactin (1j8i.1.A in the rcsb protein database) is the template used for the homology modelling of P10. The homology model starts from P10 residue N°24 (GLU, Glutamic acid) and ends with the residue N° 102 (SER, Serine). The NMR spectroscopy was used to determine the experimental structure of 1j8i.1.A [62]. The blue color show regions of the model where P10 was well modeled and orange regions where P10 was poorly modeled. The chemokine interleukin-8-like domain of the model starts with P10 amino acid at position N°27(HIS, Histidine) and ends with amino acid at position N°86 ((LEU, Leucine). This region includes both well-modeled (blue) and poorly-modeled (orange) sections

**Fig. 5:**
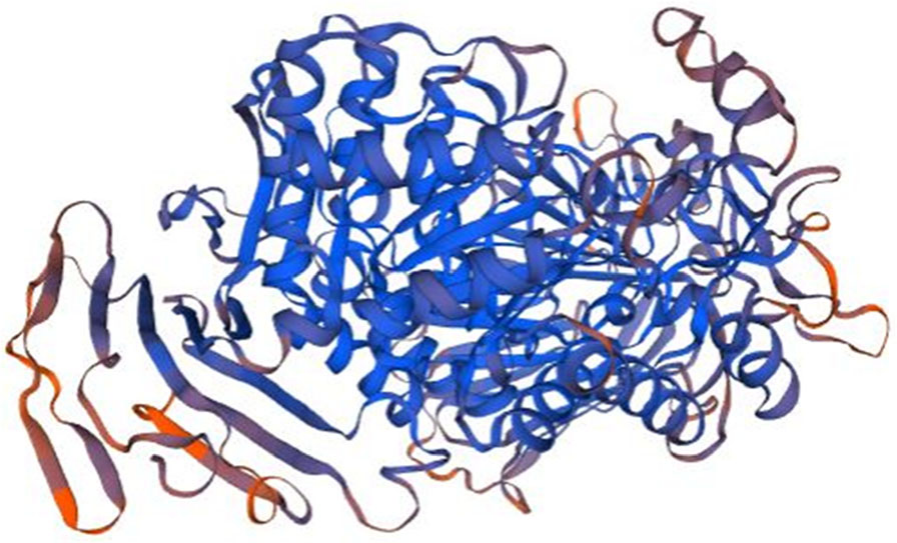
Homology model of P11. Maltase-glucoamylase, intestinal (3top.1.A in the rcsb protein database) is the template used for the homology modelling of P11. The X-RAY diffraction 2.9 Å was used to determine the experimental structure of 3top.1.A [63]. The homology model starts from P11 residue N°922 (LYS, Lysine) and ends with the residue N° 1804 (PHE, Phenylalanine). The P-type trefoil domain (amino acid N°51–962), galactose mutaros domain (amino acid N°114–1085), and glycoside hydrolase domain (amino acid N°225–1152) are not covered in the homology model. The blue color show regions of the model where P11 was well modeled and orange regions show where P11 was poorly modeled

**Fig. 6:**
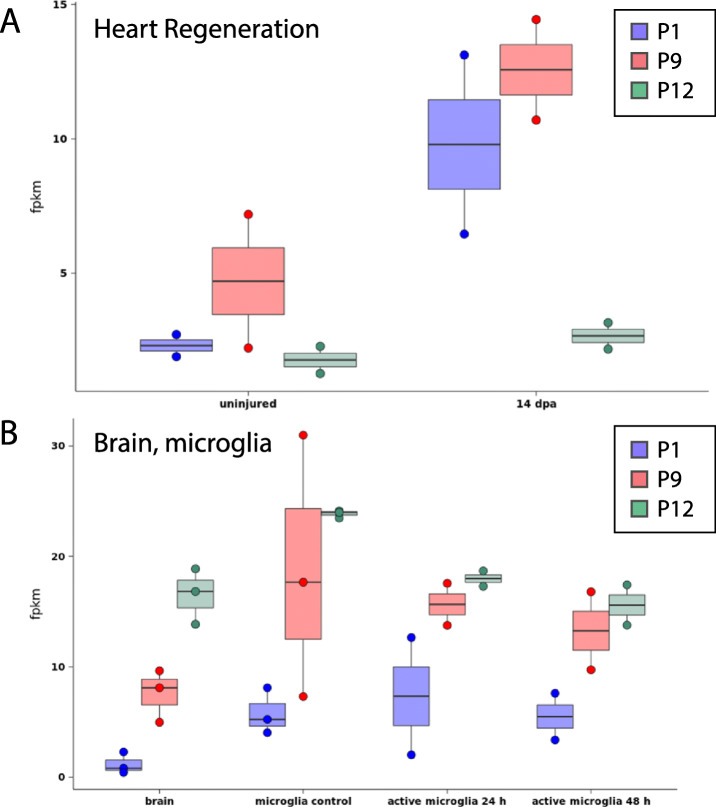
Expression level of selected zebrafish genes in other published studies. Expression level of selected zebrafish genes (P1, P9, and P12) in other published RNA-seq datasets of (**a**) zebrafish heart regeneration [64], and (**b**) zebrafish brain microglia [52] using the Zf Regeneration Database (www.zfregeneration.org, [65]). The y-axis indicates the normalized transcript level expressed as fpkm (fragments per kilobase of exon per million reads). On the x-axis is the different experimental conditions. (A,  dpa =  days post injury. B, active microglia indicates responding to acute damage, h = hours after acute damage)

A number of bioinformatic analyses were performed for the twelve genes of interest shown in Table 1 (methods summarized in Materials and Methods), and are summarized in Fig. 1. The species included in the results from these analyses are shown in Supplemental Figure 1. Protein domain and GO term were found for nine genes and largely included terms involved in immune system (Table 2). Orthologs found by sequence similarity arise from several species, mainly vertebrates (Supplemental Figure 1, Table 3); several are associated with the immune system or soluble signaling (Table 3) and the best-matched proteins are most frequently from species of fish, with occasional hits in mouse or human (Table 4). Overall, the results found for the sequence similarity and best-matched ortholog approach are consistent with the results found with the protein domain and gene ontology (GO) term approach (Tables 2, 3, 4). The three dimensional structure of the protein, or lack thereof, is known to determine protein function [56]. Of the genes studied here, two of these (P4 and P12 (*pho*)) are predicted to have greater than 50% disordered amino acids, and thus are likely to code for unstructured proteins **(**Supplemental Figure 2). We predicted three-dimensional (3D) structure using homology modeling (Table 5, Figs. 2, 3, 4, 5 and 6). The results are consistent with sequence similarity and protein domain/GO results for several genes of interest. In addition, structural similarity was informative for genes that did not return results with previous analyses (e.g. P2, P7, and P12). Synteny analysis compared to human and mouse genome returned results for only one gene (P4, with hit in human genome, Supplemental Figure 3), though based on sequence comparison this gene did not align with the candidate gene in the identified human chromosomal region. Comparison to other vertebrate species with described capacity for tissue regeneration (*Ambystoma mexicanum, Xenopus laevis, Xenopus tropicalis and Cynops pyrrhogaster*) returned putative orthologs of several of these genes (Table 6 and Supplemental Table 1) indicating that they may have conserved function across these species. More detailed descriptions of findings regarding P1-P12 are provided next.

**Table 2:** Protein domain and gene ontology (GO) term

Gene ID^a^	Protein domains	Biological process	Molecular function
P1	Protein kinase and CARD^b^ domain	Protein phosphorylation, Regulation of apoptotic process,	Protein kinase,
		Oligodendrocyte development	ATP binding
P2	*none*	*none*	*none*
P3	Immunoglobulin-like	Cell adhesion,	*none*
		Viral entry into host cell	
P4	Ribonuclease E/G	*none*	*none*
P5	MGC-24^c^ and Mucin15	*none*	*none*
P6	*none*	*none*	*none*
P7	*none*	*none*	*none*
P8	Immunoglobulin-like	*none*	*none*
P9	Urotensin II	Regulation of blood pressure,	Hormone
		Regulation of blood vessel diameter	
P10	Chemokine interleukin-8-like	Immune response	chemokine
P11	P-type trefoil, Galactose mutarotase,	Carbohydrate metabolic process	Hydrolyzing O-glycosyl compounds,
	Glycoside hydrolase		Carbohydrate binding,
			N-6 Adenine-specific DNA methylases
P12	Coiled coil	Neuromast regeneration	*none*

The protein domains and gene ontology (GO) terms found to be associated with the 12 predicted zebrafish proteins of interest

^a^Gene ID: P1 to P12 correspond to the symbol used for each predicted protein subjected to bioinformatics analysis

^b^*CARD* caspase activation and recruitment domain

^c^*MGC-24* Multi-glycosylated core protein 24

**Table 3:** Orthologs and their species of origin identified by amino acid sequence similarity using EGGNOG

Gene ID^a^	Ortholog ID	Function	Evalue^b^	Species
P1	ENSLACG00000022667	Protein tyrosine kinase	1.23e-200	*Latimeria chalumnae*
	MOS	v-mos Moloney murine sarcoma viral oncogene homolog	1.86e-27	*Xenopus (Silurana) tropicalis*
	BLK	B lymphoid tyrosine kinase	1.03e-11	*Takifugu rubripes*
	Mst1r	Macrophage stimulating 1 receptor	2.07e-7	*Mus musculus*
	CSF1R	Colony stimulating factor 1 receptor	5.21e-4	*Xenopus (Silurana) tropicalis*
P2	JGI99580	Unknown	6.68e-259	*Branchiostoma floridae*
P3	ENSGMOG00000016627	Unknown	1.5e-127	*Gadus morhua*
	ENSLACG00000005016	Immunoglobulin V-set domain	3.08e-10	*Latimeria chalumnae*
	PDGFRB	Growth factor receptor	6.45e-7	*Xenopus (Silurana) tropicalis*
	NPHS1	Nephrosis 1, congenital, Finnish type (nephrin)	1.97e-5	*Xenopus (Silurana) tropicalis*
	LOC414035	Lachesin	9.06e-5	*Apis mellifera*
P4	BASP1	Unknown	1.63e-5	*Oryzias latipes*
P5	ENSXMAG00000002763	Unknown	7.04e-17	*Xiphophorus maculatus*
	JGI72098	SH3	2.17e-4	*Phanerochaete chrysosporium*
	PTPRA	Protein tyrosine phosphatase, receptor type, A	8.33e-4	*Xenopus (Silurana) tropicalis*
P6	ARC2	CD46 molecule, complement regulatory protein	8.30e-4	*Xenopus (Silurana) tropicalis*
P7	ENSXMAG00000014998	Unknown	9.61e-44	*Xiphophorus maculatus*
P8	ENSLACG00000014033	CD84 molecule	1.05e-112	*Latimeria chalumnae*
	ENSXMAG00000015872	Lymphocyte antigen 9	2.03e-77	*Xiphophorus maculatus*
	ENSGALG00000007355	Immunoglobulin V-set domain	1.22e-09	*Latimeria chalumnae*
	CEACAM6	Carcinoembryonic antigen-related cell adhesion molecule	1.41e-09	*Takifugu rubripes*
	HMCN1	Hemicentin	3.28e-06	*Xenopus (Silurana) tropicalis*
P9	ENSXMAG00000013611	Urotensin II	2.24e-70	*Xiphophorus maculatus*
P10	ENSG00000143185	Chemokine (C motif) ligand	3.22e-14	*Gorilla gorilla*
	ENSXMAG00000019244	Small cytokines (intecrine/chemokine), interleukin-8 like	3.86e-6	*Xiphophorus maculatus*
P11	GANAB	Glucosidase, alpha	1.38e-307	*Xenopus (Silurana) tropicalis*
P12		No orthologs found		

Orthologs found for the studied genes using the protein sequence similarity approach EggNOG 4.5.1 [55]

^a^Gene ID: P1 to P12 correspond to the symbol used for each predicted protein subjected to bioinformatics analysis

^b^The Expect value (E-value) or random background noise is the number of hits one can “expect” to see by chance when searching a database of a particular size (https://blast.ncbi.nlm.nih.gov). The lower the E-value, or the closer it is to zero, the more “significant” the match is

**Table 4:** Best-matched orthologs and their species of origin identified using SmartBLAST protein sequence analysis

Gene ID^a^	Accession ID	Orthologs	Evalue^b^	Query cover^c^	Identity^d^	Species
P1	NP_003812.1	Receptor-interacting serine/threonine-protein kinase 2 isoform 1	2.00e-39	94%	27.54%	*Homo sapiens*
	NP_620402.1	Receptor-interacting serine/threonine-protein kinase 2 isoform 1	6.00e-37	89%	28.74%	*Mus musculus*
P2	XP_005164418.2	Uncharacterized protein LOC101885950	0.00	95%	54.14%	*Danio rerio*
	XP_017210637.2	Uncharacterized protein LOC108179149	2.00e-164	79%	37.53%	*Danio rerio*
	XP_021326567.1	Uncharacterized protein LOC101885087	5.00e-151	74%	37.47%	*Danio rerio*
P3	XP_005166230.1	Uncharacterized protein LOC100136852 isoform X2	0.00	100%	54%	*Danio rerio*
	XP_016100849.1	PREDICTED: uncharacterized protein LOC107561032 isoform X3	1.00e-113	98%	58.82%	*Danio rerio*
	NP_001076332.2	Junctional adhesion molecule 3b	2.00e-02	33%	29.41%	*Danio rerio*
P4	XP_026123653.1	Uncharacterized protein LOC113106193 isoform X1	4.00e-177	100%	62.04%	*Carassius auratus*
	XP_016389660.1	PREDICTED: cell surface glycoprotein 1-like isoform X4	1.00e-173	100%	64.76%	*Sinocyclocheilus rhinocerous*
	XP_016333309.1	PREDICTED: serine-aspartate repeat-containing protein I-like isoform X1	2.00e-165	100%	63.72%	*Sinocyclocheilus anshuiensis*
	XP_016105136.1	PREDICTED: calphotin-like	3.00e-164	100%	62.79%	*Sinocyclocheilus grahami*
P5	ROL44899.1	Hypothetical protein DPX16_9111	6.00e-121	100%	63.40%	*Anabarilius grahami*
	XP_016143106.1	PREDICTED: uncharacterized protein LOC107596800	9,00e-115	100%	63.19%	*Sinocyclocheilus grahami*
	XP_016395950.1	PREDICTED: uncharacterized protein LOC107729778 isoform X2	5.00e-113	100%	62.50%	*Sinocyclocheilus rhinocerous*
	XP_018973499.1	PREDICTED: uncharacterized protein LOC109104670 isoform X2	3.00e-110	100%	61.69%	*Cyprinus carpio*
P6	XP_016397186.1	PREDICTED: cell wall protein RTB1-like	1.00e-122	91%	54.81%	*Sinocyclocheilus rhinocerous*
	XP_016343246.1	PREDICTED: mucin-5 AC-like	2.00E-122	91%	55.03%	*Sinocyclocheilus anshuiensis*
	XP_016091956.1	PREDICTED: mucin-5 AC-like	3,00E-106	91%	51.01%	*Sinocyclocheilus grahami*
	XP_016124548.1	PREDICTED: cell wall protein DAN4-like	6,00E-105	92%	52.30%	*Sinocyclocheilus grahami*
P7	RXN26987.1	Hypothetical protein ROHU_020440	9,00E-65	100%	87.88%	*Labeo rohita*
	KTG33652.1	Hypothetical protein cypCar_00001489	2,00E-64	100%	87.88%	*Cyprinus carpio*
	XP_026090693.1	Uncharacterized protein LOC113064245	2,00E-63	100%	86.87%	*Carassius auratus*
	ROL47558.1	Hypothetical protein DPX16_13273	6,00E-63	100%	86.87%	*Anabarilius grahami*
	KAA0720020.1	Hypothetical protein E1301	5,00E-58	100%	78.43%	*Triplophysa tibetana*
P8	XP_009294219.1	uncharacterized protein si:ch211-239 m17.1 isoform X4	2,00E-141	93%	98.48%	*Danio rerio*
P9	KTG45257.1	Hypothetical protein cypCar_00011656	7,00E-90	95%	85.03%	*Cyprinus carpio*
	ROL51783.1	Hypothetical protein DPX16_19302	2.00e-88	82%	94.49%	*Anabarilius grahami*
	TRY88805.1	Hypothetical protein DNTS_015019	4,00E-87	100%	77.27%	*Danionella translucida*
P10	NP_001108533.1	Chemokine (C-X-C motif) ligand 32b, duplicate 1 precursor	5,00E-10	71%	35.16%	*Danio rerio*
	NP_003166.1	Cytokine SCM-1 beta precursor	5,00E-08	68%	27.91%	*Homo sapiens*
	NP_032536.1	Lymphotactin precursor	1,00E-05	75%	27.27%	*Mus musculus*
	NP_002986.1	Lymphotactin precursor	3,00E-07	68%	27.91%	*Homo sapiens*
	NP_067418.1	C-C motif chemokine 8 precursor	2,00E-05	67%	32.61%	*Mus musculus*
P11	XP_016428050.1	Maltase-glucoamylase, intestinal isoform 2	0.00	98%	57.17%	*Homo sapiens*
	NP_001074606.1	Sucrase-isomaltase, intestinal	0.00	99%	55.67%	*Mus musculus*
P12	AAI28789.1	Zgc:165381 protein	0.00	26%	100%	*Danio rerio*

^a^Gene ID: P1 to P12 correspond to the symbol used for each predicted protein subjected to bioinformatics analysis

^b^E value: The Expect value (E-value) is the number of hits one can “expect” to see by chance when searching a database of a particular size (https://blast.ncbi.nlm.nih.gov). The lower the E-value, or the closer it is to zero, the more “significant” the match is

^c^Query cover is the percentage of the query’s sequence (zebrafish gene of interest) that overlaps the subject’s sequence (returned orthologs)

^d^Identity is calculated as the percentage of characters (amino acid) within the covered part of the query that are identical

**Table 5:** Protein structure analysis

Gene ID^a^	Template ID^b^	Function	GMQE^c^	Coverage^d^	Identity^e^
P1	6fu5.1.B	Receptor-interacting Serine/threonine-protein kinase 2	0.34	55%	30.50%
	3sd0.1.A	Glycogen synthase kinase-3 beta	0.35	58%	19.26%
	4xlv.1.A	Insulin receptor	0.32	51%	23.19%
P2		No templates were found matching the sequence			
P3	3of6.1.A	T cell receptor beta chain	0.38	70%	19.31%
	5fhx.1.C	Antibody fragment light chain	0.38	72%	14.35%
	6bpc.1.B	Monoclonal antibody 4F7 Fab heavy chain	0.34	69%	15.50%
P4		No templates were found matching the sequence			
P5		No templates were found matching the sequence			
P6		No templates were found matching the sequence			
P7		No templates were found matching the sequence			
P8	6e56.1.B	Antibody pn132p2C05	0.49	90%	21.93%
	5n4g.1.A	Heavy Chain	0.49	93%	23.08%
P9		No templates were found matching the sequence			
P10	1j8i.1.A	Lymphotactin	0.42	60%	30.26%
	1ncv.1.B	Monocyte chemoattractant protein 3	0.41	59%	32.43%
	5eki.5.A	C-C motif chemokine 21	0.40	55%	27.54%
P11	3top.1.A	Maltase-glucoamylase, intestinal	0.45	49%	59.66%
	3lpo.1.A	Sucrase-isomaltase	0.44	48%	57.04%
	5nn3.1.A	Lysosomal alpha-glucosidase	0.38	46%	41.65%
P12		No templates were found matching the sequence			

Protein structure analysis using SWISS-MODEL [57]

^a^Gene ID: P1 to P12 correspond to the symbol used for each predicted protein subjected to bioinformatics analysis

^b^Template ID: 3D structure found that modeled the zebrafish protein of interest

^c^GMQE: Global Model Quality Estimation [58], which is the quality estimation of the model taking account properties from the target–template alignment and the template search method. GMQE is a number between 0 and 1. Higher numbers indicate higher reliability. A cut-off of GMQE> 0.3 was applied

^d^Coverage: The percentage of the query’s sequence (P1 to P12) that overlaps the Template sequence

^e^Identity is the percentage of characters (amino acid) within the covered part of the query that are identical. Template ID correspond to the name of the template (Ortholog) in the Protein Data Bank (https://www.rcsb.org/ [59];)

**Table 6:** Othologs found in the species *Agmbystoma mexicanum*, *Xenopus laevis, Xenopus tropicalis* and *Cynops pyrrhogaster*

Gene ID^a^	Accession ID	Function^b^	E Value^c^	Query cover^d^	Identity^e^	Species
***A.*** ***Ambystoma mexicanum***						
P1	AIW46262.1	Receptor tyrosine kinase-like orphan receptor 2	1.00e-09	40%	22.90%	*Ambystoma mexicanum*
**B.** ***Xenopus***						
P1	XP_018112660.1	Threonine-protein kinase 2-like isoform X1	3.00e-32	39%	32.24%	*Xenopus laevis*
P3	XP_004916146.1	Cell adhesion molecule 1 isoform X2	3.00e-02	56%	23.78%	*Xenopus tropicalis*
P5	XP_018101840.1	Uncharacterized protein	2.00e-32	58%	40.11%	*Xenopus laevis*
P8	XP_004919377.2	CD48 antigen	7.00e-08	99%	28.97%	*Xenopus tropicalis*
P9	KAE8621564.1	Hypothetical protein XENTR_v10004882	2.00e-06	23%	45.95%	*Xenopus tropicalis*
P10	XP_018120302.1	Cytokine SCM-1 beta-like	1.00e-07	64%	36.59%	*Xenopus laevis*
P11	XP_012818887.1	Sucrase-isomaltase, intestinal	0.00	99%	58.89%	*Xenopus tropicalis*
**C.** ***Cynops pyrrhogaster***						
P1	BAB44154.1	Insulin-like growth factor I receptor	2.00e-10	44%	23.17%	*Cynops pyrrhogaster*

BLASTP BLOSUM45 was used to find distantly related proteins in the shown species

^a^Gene ID: Corresponds to the symbol used for each predicted zebrafish protein subjected to bioinformatics analysis, the query. Only those with hits are shown

^b^Function: Corresponds to the function associated with the ortholodg found for each gene

^c^E Value: The Expect value (E-value) or random background noise is the number of hits one can “expect” to see by chance when searching a database of a particular size (https://blast.ncbi.nlm.nih.gov). The lower the E-value, or the closer it is to zero, the more “significant” the match is

^d^Query cover: The percentage of the query’s sequence (zebrafish gene) that overlaps the subject’s sequence (returned ortholog)

^e^Identity: The percentage of amino acids within the covered part of the query that are identical between the query and the returned ortholog

#### **P1 (si:dkey-181f22.4)**

The gene coding for P1 (*si:dkey-181f22.4*) is located on zebrafish chromosome 7 and is predicted to have exon/intron structure coding for a predicted 513 amino acid protein (Table 1). Protein domain and gene ontology (GO) term returned predicted “protein kinase domain” and “Caspase Activation and Recruitment (CARD) domain” (Table 2). The CARD domain is known to function in innate immunity, particularly in inflammation and the regulation of apoptotic process (Table 2, [66–69]). Amino acid sequence similarity analysis returned several kinases associated with immune function, and suggested that this gene may code for a receptor tyrosine kinase (Table 3). The best-matched ortholog analysis returned “Receptor-interacting serine/threonine-protein kinase 2 isoform 1” in both human and mouse (Table 4). Of note, human RIPK2 has been described to contain a C-terminal CARD domain [70–72]. In comparison to other selected species (Table 6), P1 returned receptor tyrosine kinase-like orphan receptor 2 (Axolotl), Threonine-protein kinase 2-like isoform X1 (*Xenopus*), and insulin-like growth factor receptor as well as receptor tyrosine kinase-like orphan receptor 2 (Salamander). Structure prediction (Table 5, Fig. 2) strongly indicated a kinase domain/function for P1.

The results strongly indicate that P1 has a kinase domain that may be activated by interactions with other proteins via the CARD domain, and this function may be acting in concert with receptor activity. Interestingly, the CARD domain of human RIPK2 facilitates interaction with NOD-like receptors [73, 74]. Collectively, these results indicate that zebrafish P1 may have orthologous function to human RIPK2. However, the amino acid substrate of phosphorylation (tyrosine vs. serine/threonine) by zebrafish P1 is not yet clear, as both classes of kinases were indicated in the hits.

#### **P2 (si:ch73-112 l6.1)**

The gene for P2 (*si:ch73-112 l6.1*) is located on zebrafish chromosome 21 and codes for a predicted 1025 amino acid protein (Table 1). Protein stability analysis (Supplemental Figure 2) indicates P2 is a structured protein, but with a large disorded domain. Such disordered regions often indicate a protein-protein binding interface [56]. However, collective analyses were largely uninformative for P2. For example, no protein domains nor GO terms were returned (Table 2). A putative ortholog with unknown function from *Branchiostoma floridae* was returned based on amino acid sequence similarity (Table 3), and three uncharacterized zebrafish genes were returned as best-matched orthologs (Table 4).

#### **P3 (zgc:174863)**

The gene for P3 (*zgc:174863*) is located on zebrafish chromosome 6 and codes for a predicted 290 amino acid protein (Table 1). Protein domain and GO terms indicate an immunoglobulin-like domain, which are present in proteins involved in cell adhesion (Table 2). Consistent with this, sequence similarity analysis revealed 5 proteins from 4 species, several of which contain immunoglobulin folds (Table 3). Protein structure analysis (Table 5, Fig. 3) further indicated that the predicted protein contains immunoglobulin-like domains as it was resonably modeled by the T cell receptor beta chain in regions containing immunoglobulin folds (Fig. 3). Collectively, these results suggest that P3 could be a cell membrane receptor possibly involved in cell adhesion. In support of this, comparison to *Xenopus tropicalis* returned a predicted ortholog with putative cell adhesion function (Table 6). In addition, several hits for P3 were found by amino acid similarity in *Xenopus tropicalis*, *Apis mellifera*, *Gadus morhua*, and *Latimeria chalumnae* (Table 3), and based on phylogenetic relationships of these species (Supplemental Figure 1), it seems possible that the funciton of the gene coding for P3 was evolutionarily conserved in these species.

#### **P4 (si:dkey-56 m19.5)**

The gene coding for P4 (*si:dkey-56 m19.5*) is located on zebrafish chromosome 7 and codes for a predicted 526 amino acid protein (Table 1). As noted above, P4 is predicted to be a disordered protein (Supplemental Figure 2). Many intrinsically disordered proteins evolve rapidly [75–78], and therefore, predicting a function for P4 is difficult based on amino acid sequence. Accordingly, analyses based on sequence similarity were overall minimally informative. An associated protein domain (Ribonuclease E/G) was returned for P4 (Table 2) and a possible ortholog (Brain abundant, membrane attached signal protein 1, BASP1) with unknown function in *Oryzias latipes* was a hit based on amino acid sequence similarity (Table 3). P4 returned four best-matched orthologs from other species, but these genes had widely varying predicted functions (Table 4). Protein structure analysis was uninformative for P4 (Table 5).

Synteny analysis indicated that the gene coding for P4 lies in a syntenic region with human genome on human chromosome 16 (Supplemental Figure 3). The gene for P4 is flanked by several neighboring genes that have apparent orthologs in human, and based on the orientations and locations of the neighboring genes in the two species, the gene for P4 lies in a relative location similar to human TERB1. However, using NCBI BLASTP to compare sequences of zebrafish P4 and human TERB1 (with any scoring matrix) found no signficant similarity between these two genes, therefore failing to provide evidence of orthology of these genes. Therefore, we consider that the gene coding for P4 could have been gained in zebrafish or lost in humans. Interestingly, several possible orthologs in various species of fish were returned for P4 (Table 4).

#### **P5 (si:ch211-105j21.9)**

Protein domain and GO term returned MGC-24 and Mucin15 domain (Table 2) for P5 (*si:ch211-105j21.9*). Amino acid sequence similarity returned three hits from three different species for genes with unknown and varying functions (Table 3), but best-matched orthologs (Table 4), as well as protein structure analysis, was uninformative. Although a hit was found in *Xenopus laevis* (Table 6), the protein has unknown function.

#### **P6 (si:ch73-248e21.7)**

P6 (*si:ch73-248e21.7*) did not return any hits for GO terms, but a putative complement regulatory protein from *Xenopus tropicalis* was identified as a hit by sequence similarity analysis (Table 3). Best-matched orthologs were found in four *Sinocyclocheilus* species of fish, two of which were Mucin 5AC_like proteins and two of which were cell wall-like proteins (Table 4). However, other analyses proved uninformative.

#### **P7 (si:ch211-191j22.3)**

Analyses for P7 were largely uninformative, though there were hits in some of these analyses indicating unknown, uncharacterized, or hypothetical proteins in six different fish species (Table 3, Table 4) their meaning was not interpretable.

#### **P8 (LOC100535303)**

Protein domain/GO term results suggest P8 contains immunoglobulin-like domain. This was further indicated by the amino acid sequence similarity results (Table 3), protein structure results (Table 5), and the putative “CD48 antigen” orthologue identified in *Xenopus tropicalis* (Table 6).

#### **P9 (urp1)**

The gene coding for P9 was previously annotated as *urp1*, suggesting that putative urotensin function is already recognized. Consistent with this, protein domain/GO term and amino acid sequence similarityreturned results for P9 indicating urotensin function (Table 2 and Table 3), which is involved in regulation of vasculature diameter. Specifically, Urotensin II is a secreted mediator known to function in vasoconstriction of blood vessel diameter (Table 2, [79–81]). However, similar structures were not identified in our analyses (Table 5).

#### **P10 (xcl32a.1)**

The gene for P10 (*xcl32a.1*) is located on zebrafish chromosome 2 and is predicted to encode a protein of only 126 amino acids (Table 1). The protein domains/GO term search returned chemokine interleukin-8-like, which functions in immune response (Table 2). Other analyses also indicated that P10 is likely a cytokine/chemokine (Table 3, Table 4, Table 5, Table 6). The predicted amino acid length of P10 is consistent with short amino acid chains seen in cytokines/chemokines. Consistent with this function, regions of P10 were well modeled by regions of the chemokine Lymphotactin’s interleukin-8-like domain (Fig. 4).

#### **P11 (si:ch211-287n14.3)**

Collectively, results for P11 indicate that it could be an enzyme involved in carbohydrate metabolism (Table 2, Table 3, Table 4, Table 5, and Table 6). P11 could be well modeled by human intestinal maltase-glucoamylase (Table 5, Fig. 5), as well as sucrase-isomaltase and lysosomal alpha-glucosidase (Table 5). However, the predicted functional domains found previosly (P-type trefoil, galactose mutarose, and glycoside hydrolase domains, Table 2), were not covered in the homology model of maltase-glucoamylase. The domain P-type trefoil, found for P11 (Table 2), is found in several secreted proteins associated with mucins [82–84], many of which are involved in the response to gastrointestinal mucosal injury and inflammation [85], though the function of such a secreted protein in the CNS during tissue regeneration is not clear; perhaps it could be involved in extracellular matrix degradation.

#### **P12 (pho)**

The gene encoding P12 (*pho*) is located on zebrafish chromosome 5 and encodes a large predicted protein of 2798 amino acids (Table 1). Interestingly, P12 (*pho*) has been previously described to be required for the regeneration of zebrafish neuromasts [86], which are sensory patches located along the zebrafish body, but its function has not been studied otherwise. The coiled coil domain found in the protein domain/GO term analysis (Table 2) was described previously [86]. In addition, we find that P12 is predicted to have more than 50% of the amino acids disordered, and is therefore is likely an unstructured protein **(**Supplemental Figure 2). Since P12 is a disordered protein, this is likely the reason that other analyses did not prove informative (Table 3, Table 4, Table 5, Table 6). Many studies have shown that disordered proteins evolve more rapidly than structured proteins [75–78] and the disordered region of the protein drives this rapid evolution [77]. In addition, large proteins with coiled-coil domains appear to have functions in cell structure [56]. In spite of the predicted disordered structure, the previously cited study [86] found evidence for an ATPase and transmembrane domain; however, our analyses did not reveal these features. Given that P12 is reported to be required for neuromast regeneration in zebrafish [86], we considered that a syntenic relationship might be identified in genomes of other species known to have robust regenerative abilities. However, our synteny analyses did not return predicted syntenic regions compared to *Ambystoma mexicanum*, *Xenopus laevis*, *Xenopus tropicalis*, *Cynops pyrrhogaster* (not shown).

#### **Comparison to other published RNA-seq datasets**

We were interested in determining to what extent transcripts mapping to some select genes might be shared in other zebrafish tissue/cells such as regenerating tissue such as heart [64], in resting microglia [52], and in microglia responding to acute damage [52]. We focused this comparison on P1, P9, and P12 because P1 had particularly informative analyses above (indicating kinase function), and P9 and P12 might have novel functions in regeneration. Interestingly, transcripts for both P1 and P9 were increased in regenerating heart tissue samples compared to uninjured (Fig. 6a). Transcripts mapping to P1 appeared slightly more abundant in resting microglia compared to other brain cells, but levels did not change significantly in microglia responding to acute damage (Fig. 6b). Since P1 was enriched in microglia in our study [30], which sampled microglia/macrophages during retinal regeneration, it is possible that expression and function of this putative kinase (P1) are upregulated during tissue regeneration. Transcripts for P9 gene were present in microglia in the zebrafish brain, both in resting state and in response to acute brain damage (Fig. 6b), though they did not appear to change significantly in such conditions. Thus, it is possible that P9 is a mediator produced by microglia/macrophages that acts on the local vasculature to control blood pressure locally and perhaps this function is upregulated during tissue regeneration.

Examining expression levels of P12 did not demonstrate any apparent upregulation of P12 in regenerating heart compared to the very low transcript levels in uninjured heart tissue (Fig. 6a). However, P12 expression was observed in resting microglia from zebrafish brain, and the expression of P12 appeared to be reduced in context of microglial acute damage response [52] (Fig. 6b). This expression pattern, in combination with our dataset indicating expression by microglia/macrophages during retinal regeneration, suggests that P12 (*pho*) may have function in restoring and/or maintaining a “resting” microglial/macrophage state. However, such a hypothesis will require experimental testing.

We next examined a published RNA-seq dataset representing zebrafish macrophages responding to *M. marinum* infection [87], to determine if the genes of interest were also differentially expressed in zebrafish macrophages responding to microbial infection. Interestingly, although transcripts were detected in the Rouget et al. study for ten out of twelve of the genes, only one of these (P6, *si:ch73-248e21.7*, which may have complement regulatory function based on the results describbed above) was found to be differentially expressed in macrophages from infected fish compared to uninfected fish based on the authors’ cut-off criteria of Log2FC > =1, p-adj < 0.05 (Table 7). This supports the idea that these genes could comprise part of a unique transcriptome that is expressed in microglia/macrophages during tissue regeneration compared to that in response to microbial infection.

**Table 7:** Expression of zebrafish genes pertaining to P1-P12 in macrophages responding to microbial infection

Gene ID^a^	Zebrafish Symbol^b^	Ensembl ID	DE^c^ in Macrophages responding to *M. marinum* infection?
P1	si:dkey-181f22.4	ENSDARG00000105643	*ND*
P2	si:ch73-112 l6.1	ENSDARG00000093126	No
P3	zgc:174863	ENSDARG00000099476	*ND*
P4	si:dkey-56 m19.5	ENSDARG00000068432	No
P5	si:ch211-105j21.9	ENSDARG00000097845	No
P6	si:ch73-248e21.7	ENSDARG00000096331	Yes
P7	si:ch211-191j22.3	ENSDARG00000095459	No
P8	LOC100535303	ENSDARG00000071653	No
P9	urp1	ENSDARG00000093493	No
P10	xcl32a.1	ENSDARG00000093906	No
P11	si:ch211-287n14.3	ENSDARG00000093650	No
P12	pho	ENSDARG00000035133	No

The twelve genes of interest were examined in the RNA-seq dataset from Rouget et al.*,* 2019 (GSE78954 and GSE68920), which examined the transcriptome of zebrafish macrophages responding to *M. marinum* infection

^a^Gene ID: Corresponds to the zebrafish gene of interest in this study

^b^Zebrafish Symbol: corresponds to the symbol attributed to each gene by the ZFIN Zebrafish Nomenclature Conventions

^c^DE: Differential Expression in zebrafish macrophages responding to infection compared to uninfected. Using the RNA-seq datasets from Rouget et al.*,* 2019, DE was based on the authors’ original criteria of logFC greater than or equal to 1, and p-adj < 0.05. “Yes” or “No” indicates that the gene was differentially expressed or not, respectively. *ND* indicates that the transcript not detected in the dataset

## Discussion

In this study, we analyzed twelve zebrafish genes with unknown function. These genes were selected from our previous transcriptome analysis of zebrafish microglia/macrophages isolated from regenerating retinal tissue [30]. We used bioinformatic analyses to analyze the twelve selected transcripts to suggest putative functions. These analyses included protein domain and gene ontology (GO) terms, amino acid similarity, predicted protein structure, and synteny comparisons. For some selected genes, we examined expression level in other published studies of gene expression in zebrafish [52, 64], and examined other published data sets involving macrophages responding to microbial infection [87] to determine if these genes might be regulated in different activation contexts.

Results for many of the genes analyzed indicate putative functions related to the immune system. Several of these functions may not be well described in fish compared to mammalian organisms. The predicted genes/predicted proteins yielding the most informative results include P1 (results strongly indicate receptor associated kinase activity), P9 (previously annotated as urp1, which results indicate urotensin-like activity), P10 (which may have chemokine activity), and P11 (which could be an enzyme involved in carbohydrate metabolism). Although only an immunoglobulin-like fold domain was revealed for P3 and P8, and a possible mucin domain for P5, these results provide at least some new insight into the structure of the predicted proteins as these domains have not been previously noted for these genes. On the other hand, our analyses did not reveal significant functional information about P2, P4, P6, P7, and P12. Given that P12 (*pho*) is predicted to be a disordered protein, our analyses do not allow us to make predictions about the function of this particular protein, though it remains of interest due to its previously indicated role in neuromast regeneration [86]. It will be interesting to determine, experimentally, if phoenix (*pho*), or any of the other genes analyzed in this work, are required for retinal regeneration.

The lack of syntenic relationships between zebrafish and mouse/human for the majority of the genes analyzed is notable, suggesting that possibly these genes were not evolutionarily retained across these species or alternatively, that these genes may have appeared in certain species [88]. For the one zebrafish gene that did have syntenic relationship identified, sequence alignment did not indicate an evolutionary relationship to the candidate gene in the syntenic region. Orthologs were identified for some, but not all, of these zebrafish genes of interest in species which are also known to regenerate damaged tissue (Axolotl, *Xenopus* and Salamander, Table 6 and Supplemental Table 1). We therefore consider that, in future work, it is important to determine if the genetic program used by microglia/macrophages during zebrafish CNS regeneration is unique on a species level. Whether such a unique genetic program is required for successful regeneration also remains to be determined.

To begin to probe this question, we examined other published RNA-seq datasets for expression patterns of the genes examined here in this work. For selected genes (P1, P9, and P12), we examined transcript abundance in samples from zebrafish regenerating heart tissue [64] and zebrafish brain microglia [52]. Both P1 and P9 showed upregulation in regenerating zebrafish heart, while P12 transcripts were apparently reduced in microglia responding to acute damage compared to resting microglia. When we examined the transcriptome of zebrafish macrophages responding to infection by the microbe *M. marinum* [87], only one of the twelve genes discussed in our work here was found to be differentially expressed in this context. It is worth considering that the samples sequenced in our study [30] compared to these other studies differ in regards to the developmental age/stage of the animal, location in the body, sample preparation, sequencing protocols, as well as other factors. However, these comparisons might still suggest that it is possible that these genes may be regulated in a tissue regeneration context rather than in response to microbial infection. Thus, it is possible that at least some of these genes comprise part of a general transcriptional program active in zebrafish microglia/macrophages responding to both tissue damage and/or infection. However, further experimental studies involving at least some of these genes (i.e. P1, which bioinformatic predictions suggest could be a kinase, and P12 (*pho)*) are likely to increase our understanding of mechanisms involved in successful tissue regeneration. Indeed, harnessing such regenerative capacity in mammals must be better informed by a more thorough functional understanding of a genetic program executed by organisms such as zebrafish, that underlies successful regeneration. Such work will also lead to a better evolutionary understanding of the vertebrate innate immune system.

## Conclusions

In this study, we have predicted putative functions for several zebrafish genes with previously unknown function. Transcripts mapping to these genes were enriched in microglia/macrophages during retinal regeneration, suggesting they could have functional importance in tissue regeneration. We identified putative orthologs of several of these genes, mainly based on functional domains, which provide informative insight into possible protein function. In addition, comparison to other RNAseq datasets suggest that most of these genes could be expressed as part of a transcriptional program expressed by microglia/macrophages during tissue regeneration. Our findings provide a foundation for future experimental work to determine the function of these genes in vivo.

## Methods

### RNAseq dataset and predicted orthology

The 3’mRNA Quant-seq experiment and differential gene expression (DEG) analysis is described in Mitchell et al.*,* 2019 [30]. This dataset is available on the Gene Expression Omnibus (GEO120467). To identify putative mouse and human orthologs of the 986 transcripts found to be enriched in *mpeg*1+ cells compared to other cell types, the DRSC integrative ortholog prediction tool (DIOPT, v 7.0, www.flyrnai.org) was employed based on the zebrafish ENSEMBL ID.

### Protein domains and gene ontology (GO) terms

The protein domains and the gene ontology (GO) terms (Biological Process and Molecular Function) were determined from the universal protein knowledgebase (UniProt, [89]) and the integrative protein signature database (InterPro, [90]). The gene ID from Ensembl (https://www.ensembl.org/, [54]) was used to extract the predicted protein sequence of the gene from the National Center for Biotechnology Information database (NCBI, https://www.ncbi.nlm.nih.gov/). The gene’s amino acid sequence was used to extract protein domains and gene ontology (GO) terms in UniProt [89] and InterPro [90].

### Sequence similarity

Two approaches were used to find orthologs for each protein based on sequence similarity, EggNOG and SmartBLAST, because these two approaches use different protein databases. The bioinformatics web-server *EggNOG 4.5.1* [55] compares the input protein sequence to the sequences available in several databases and displays the list of orthologs of the protein and the species where those orthologs are found [55]. The “default” settings of the web-server SmartBLAST (https://blast.ncbi.nlm.nih.gov/smartblast/) was used to identify the species of origin of orthologs (and paralogues within zebrafish) which were best-matched by our genes using the non-redundant protein sequence database [91].

To look for orthologs in species with described capacity for regeneration (*Ambystoma mexicanum, Xenopus laevis, Xenopus tropicalis, Cynops pyrrhogaster*), the protein sequences of zebrafish genes were compared to the NCBI database (http://blast.ncbi.nlm.nih.gov) using BLASTP with the BLOSUM45 scoring matrix and Gap Costs “Existence: 10 Extension: 3” (http://blast.ncbi.nlm.nih.gov). In addition, we used tBLASTn to identify putative unannotated orthologs in these species, and these results are reported in Supplemental Table 1.

### Structural analysis

We inferred protein disorder using default settings (5% false positive rate) of the the server *PrDOS* (http://prdos.hgc.jp/cgi-bin/top.cgi, [92]), which predicts natively disordered regions of a protein chain from its amino acid sequence. *PrDOS* returns a disorder probability for each residue. Proteins with more than 30–50% predicted disordered residues are considered disordered proteins [92].

We used the bioinformatics web-server SWISS-MODEL [57] to identify templates or homologs for our list of unknown proteins based on the predicted 3D structure of the proteins of interest (with Global Model Quality Estimation [58] or GMQE > 0.3 as cut-off). Homology modeling, or comparative protein modeling, uses an ortholog’s (template’s) experimentally-determined 3D-structure to estimate a model for the target sequence [57].

### Synteny analysis

Synteny comparisons were performed using www.ensembl.org, because this database uses the most updated genome build for zebrafish (GRCz11). The ENSEMBL ID was used to identify the gene of interest and the chromosomal region containing the gene was selected. In the Comparative Genomics menu option, synteny was selected to compare the chromosomal region of the zebrafish gene to human (GRCh38.p13) and mouse (GRCm38.p6) genomes. Only one gene of interest was found to lie in a syntenic region (P4, Supplemental Figure 3). The amino acid sequence of the zebrafish gene was compared using (BLASTP, http://blast.ncbi.nlm.nih.gov) to the candidate annotated gene found inside the syntenic region using the National Center for Biotechnology Information (NCBI) database to look for similarity and orthologs; alignment was compared with each scoring matrix in the program [93].

### Expression level in other RNA-seq datasets

We determined the expression level of selected zebrafish genes of interest in other published datasets of zebrafish heart regeneration [64] and zebrafish brain microglia [52] using the Zf Regeneration Database (www.zfregeneration.org) [65]. The gene’s symbol or ENSEMBL ID were used to plot the normalized expression level of transcripts of interest.

To probe the RNA-seq dataset from Rouget et al. [87]*,* we searched for the ENSEMBL ID of each gene of interest in the raw datasets (GSE78954 and GSE68920) to determine if transcript counts were detected. To determine if the gene was considered to be differentially expressed in macrophages responding to infection, we examined the authors’ reported results of differential expression analysis comparing transcripts from sorted uninfected vs. *M. marinum* infected macrophages from zebrafish larvae [87] (Rouget et al.*,*2019).

## Supplementary Information

**Additional file 1: **Supplemental File 1, Orthology predictions of differentially expressed genes.

**Additional file 2: **Contains Supplemental Figures 1-3 and Supplemental Table 1.

## Data Availability

The original RNAseq dataset (Mitchell et al., 2019) is available on the Gene Expression Omnibus (GEO120467).

## Abbreviations

ARC2: Amphibian RCA protein 2
ATP: Adenosine triphosphate
BASP1: Brain abundant, membrane attached signal protein 1
BLAST: Basic local alignment search tool
BLASTN: Nucleotide BLAST
BLASTP: Protein BLAST
BLK: BLK proto-oncogene, Src family tyrosine kinase
BLOSUM: Blocks Substitution Matrix
CALCB: Calcitonin related polypeptide beta
CARD: Caspase activation and recruitment domain
CD48: Cluster of Differentiation 48
CDS: Coding DNA Sequence
CEACAM6: CEA cell adhesion molecule 6
CNS: Central nervous system
CSF1R: Colony stimulating factor 1 receptor
DEGs: Differentially expressed genes
DNA: Deoxyribonucleic acid
ENSG00000143185: XCL2 = X-C motif chemokine ligand 2
ENSLACG00000005016: LOC102359650 = programmed cell death 1 ligand 1-like
ENSLACG00000022667: SI:DKEY-181F22.4 = receptor-interacting serine/threonine-protein kinase 2-like
ENSXMAG00000015872: LOC102236357 = hepatocyte cell adhesion molecule-like
FC: Fold-Change
GANAB: Glucosidase, alpha; neutral AB
GO: Gene Ontology
GRCh38: Genome Reference Consortium Human Build 38
GRCm38: Genome Reference Consortium Mouse Reference 38
GRCz11: Genome Reference Consortium *Danio rerio* Build 11
HMCN1: Hemicentin 1
HS2ST1: Heparan sulfate 2-O-sulfotransferase 1
LBD: Ligand-Binding Domain
LOC414035: Lachesin
MGC-24: Multi-glycosylated core protein 24
Mst1r: Macrophage stimulating 1 receptor (c-met-related tyrosine kinase)
MOS: MOS proto-oncogene, serine/threonine kinase
mpeg1: Macrophage-expressed gene 1 protein
NPHS1: NPHS1 nephrin
P1: si:dkey-181f22.4 = ENSDARG00000105643
P2: si:ch73-112 l6.1 = ENSDARG00000093126
P3: zgc:174863 = ENSDARG00000099476
P4: si:dkey-56 m19.5 = ENSDARG00000068432
P5: si:ch211-105j21.9 = ENSDARG00000097845
P6: si:ch73-248e21.7 = ENSDARG00000096331
P7: si:ch211-191j22.3 = ENSDARG00000095459
P8: si:ch73-256j6.2 = ENSDARG00000071653
P9: urp1 = ENSDARG00000093493
P10: xcl32a.1 = ENSDARG00000093906
P11: si:ch211-287n14.3 = ENSDARG00000093650
P12: pho = ENSDARG00000035133
PDGFRB: Platelet-derived growth factor receptor, beta polypeptide
Pho: Phoenix
PTPRA: Rotein tyrosine phosphatase, receptor type A
RNA: Ribonucleic Acid
RNA-seq, RNAseq: RNA Sequencing
3D: Three-dimensional
