# Supplementary material for: Bioinformatic analysis and functional predictions of selected regeneration-associated transcripts expressed by zebrafish microglia
Source: BMC Genomics. 2020 Dec 7;21:870. doi: 10.1186/s12864-020-07273-8 (PMC7720500; doi:10.1186/s12864-020-07273-8)
Supplement: Supplementary file 2 — Additional file 2: Contains Supplemental Figures 1-3 and Supplemental Table 1. [file 12864_2020_7273_MOESM2_ESM.pdf]

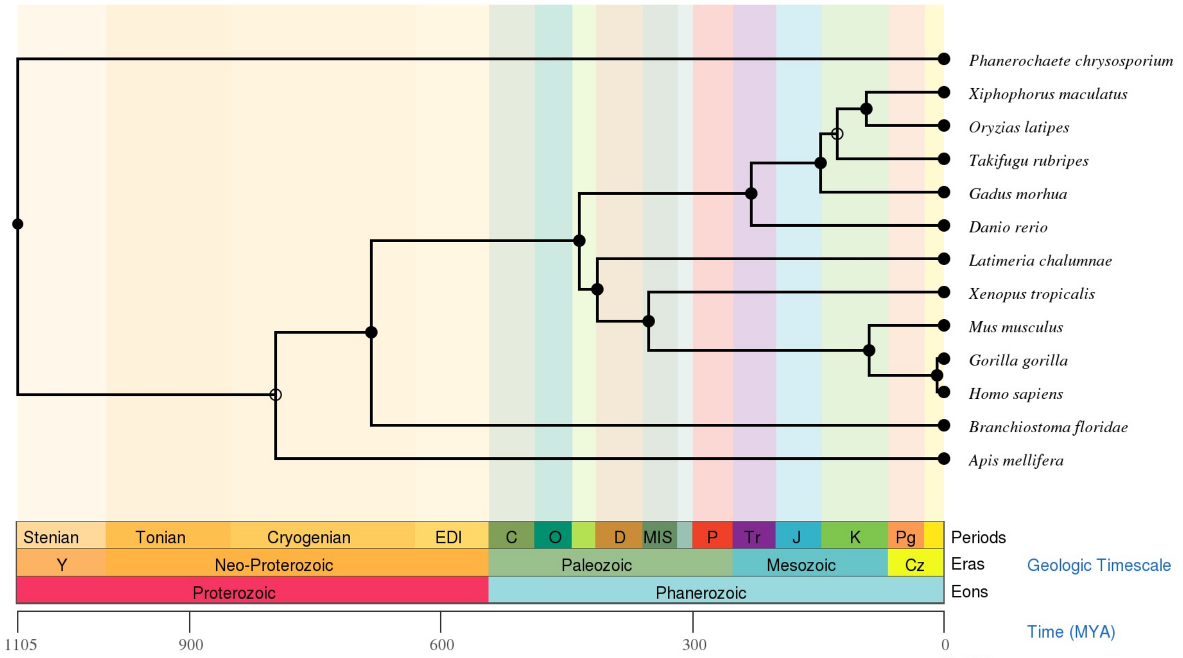

Supplemental Figure 1. Phylogenic relationship of species of origin of orthologs to the 12 zebrafish genes found by amino acid sequence similarity analysis. The web-server Phylogeny.fr (http://www.timetree.org/, Kumar *et al.,* 2017)

S. Kumar, G. Stecher, M. Suleski, and S.B. Hedges, 2017. TimeTree: a resource for timelines, timetrees, and divergence times. Molecular Biology and Evolution 34: 1812-1819, DOI: 10.1093/molbev/msx116.

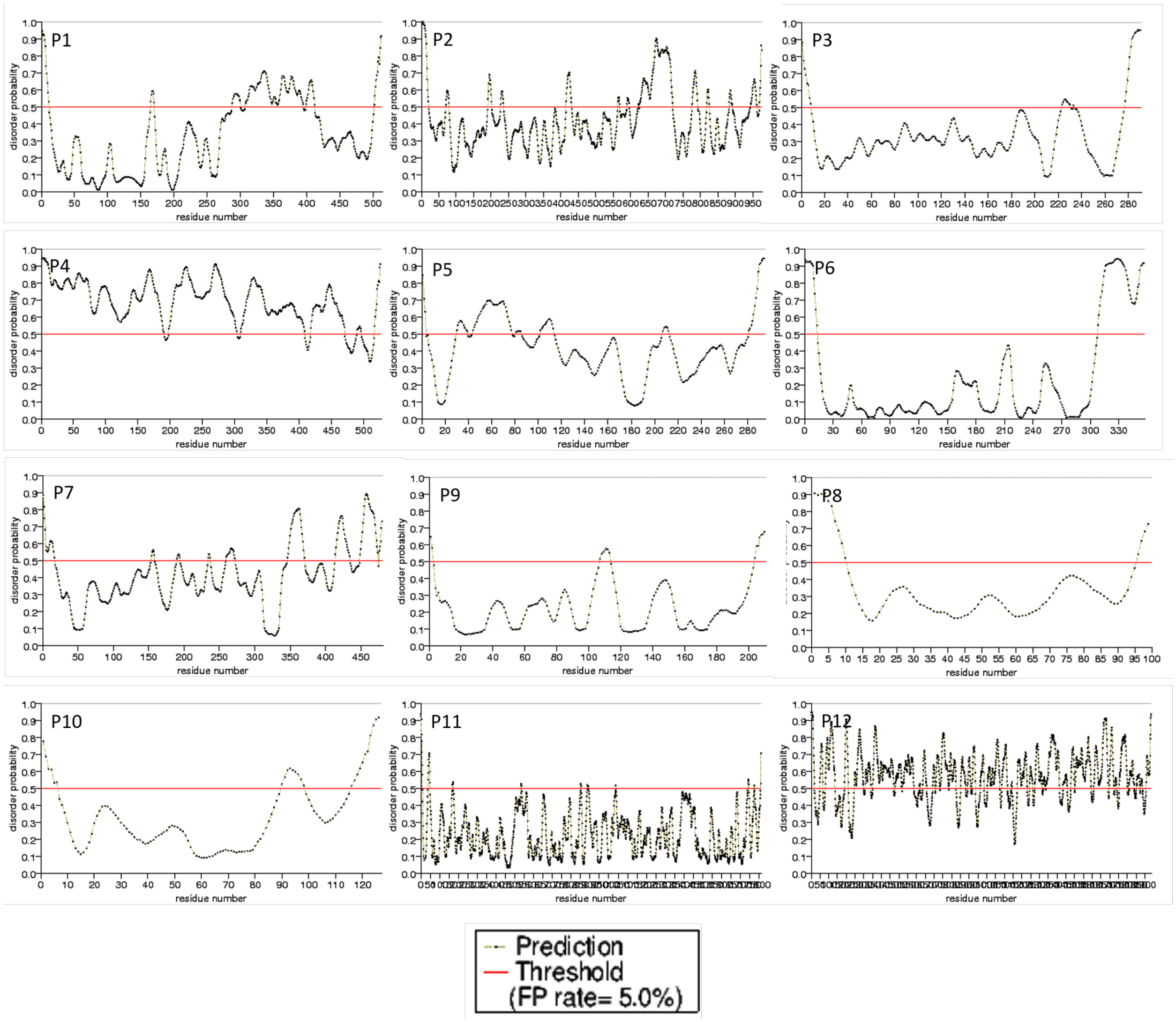

Supplemental Figure 2. Protein stability prediction. P1 to P12 correspond to the 12 zebrafish proteins of interest. On the X-axis is the residue number of the protein and the Y-axis the disorder probability. The dotted line shows the prediction and the red line the threshold (0.5). FP rate = Prediction false positive rate. Proteins that have less than 50% of their amino acids disordered after analysis are considered as disordered proteins.

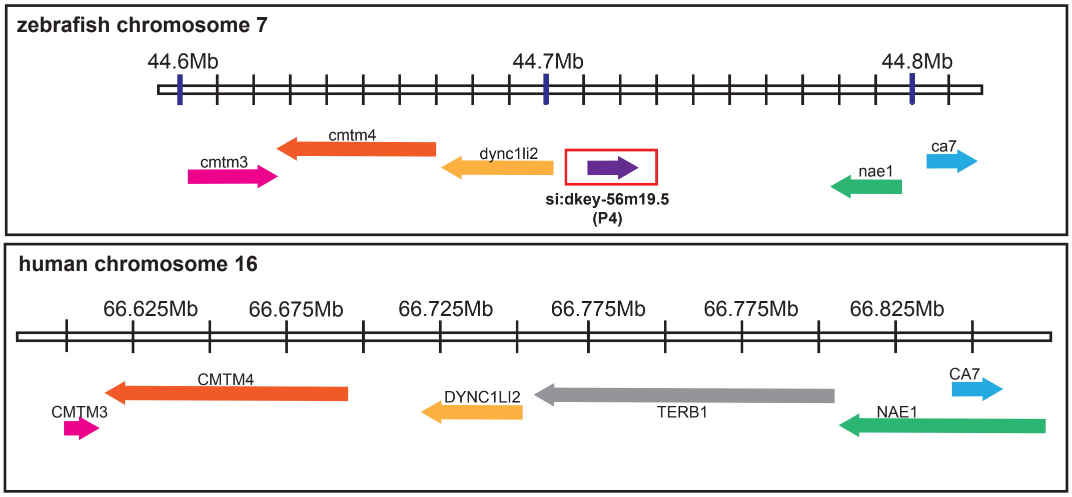

Supplemental Figure 3. Diagram of the region of zebrafish chromosome 7 containing the gene for P4 (si:dkey-56m19.5) and the corresponding region found to have syntenic relationship within human chromosome 16. Orthologous genes within the region are indicated by color matching. The location of P4 (si:dkey-56m19.5, purple arrow) is indicated by the red box, and neighboring upstream and downstream genes are shown, which are conserved in similar arrangement in humans. The gene for P4 lies in a relative location to human TERB1 making this a candidate ortholog. However, the transcriptional orientation of P4 and TERB1 is opposite of each other and, as described in the main text, there was no similarity found between their sequences.

Supplemental Table 1. Unannotated sequences found the species *Ambystoma mexicanum*, *Xenopus laevis*, *Xenopus tropicalis* and *Cynops pyrrhogaster*.

Gene ID*	Accession ID	Description**	E Value†	Query cover‡	Identity§	Species
		**A. Ambystoma mexicanum**				
P1	CN036264.1	nm_14_h19_t3_ Math Ambystoma mexicanum cDNA, mRNA sequence	6.00e-05	32%	25.71%	*Ambystoma mexicanum*
P3	CN034493.1	Math_p1_K11_T3_ Math Ambystoma mexicanum cDNA, mRNA sequence	0.33	12%	33.33%	*Ambystoma mexicanum*
P4	CN036042.1	nm_12_d13_t3_ Math Ambystoma mexicanum cDNA, mRNA sequence	0.63	9%	34.62%	*Ambystoma mexicanum*
P10	CN050376.1	v6_p4_j2_triplex5.1ld_ V6 Ambystoma mexicanum cDNA, mRNA sequence	0.04	44%	32.14%	*Ambystoma mexicanum*
P11	CN041057.1	nm_39h_g18_t3_ Math Ambystoma mexicanum cDNA, mRNA sequence	0.64	4%	25.00%	*Ambystoma mexicanum*
P12	CO779483.1	BL006D_A02 6-Day Axolotl Tail Blastema (6DAxBL) Ambystoma mexicanum cDNA 5' similar to unnamed protein product (Tetraodon nigroviridis), mRNA sequence	0.25	1%	42.86%	*Ambystoma mexicanum*
		**B. *Xenopus***				
P1	CF548705.1	AGENCOURT_15594380 NICHD_XGC_Brn1 Xenopus laevis cDNA clone IMAGE:7018578 5', mRNA sequence	2.00e-10	25%	32.84%	*Xenopus laevis*
P3	BJ074938.1	NIBB Mochii normalized Xenopus tailbud library Xenopus laevis cDNA clone XL071p01 5', mRNA sequence	0.00	49%	25.52%	*Xenopus laevis*
P7	BJ091035.1	NIBB Mochii normalized Xenopus tailbud library Xenopus laevis cDNA clone XL110o14 3', mRNA sequence	0.22	65%	29.23%	*Xenopus laevis*
P10	EB729213.1	AGENCOURT_77836029 NICHD_XGC_skin_m Xenopus laevis cDNA clone IMAGE:8643277 5', mRNA sequence	9.00e-08	53%	34.33%	*Xenopus laevis*
P11	EG578435.1	AGENCOURT_90753507 NICHD_XGC_int_m Xenopus laevis cDNA clone IMAGE:8820748 5', mRNA sequence	9.00e-123	28%	70.11%	*Xenopus laevis*
P12	BI941228.1	df25h07.y1 Wellcome CRC pRN3 St10 5 Xenopus laevis cDNA clone IMAGE:3558397 5' similar to TR:Q9YI96 Q9YI96 EF-1-ALPHA PROTEIN, mRNA sequence	0.20	1%	39.53%	*Xenopus laevis*
		**C. Cynops pyrrhogaster**				
P1	FS297191.1	Cp_aI Cynops pyrrhogaster cDNA clone Cp_aI_019_P04 3', mRNA sequence	1.00e-05	17%	31.91%	*Cynops pyrrhogaster*

P3	FS298288.1	Cp_aI Cynops pyrrhogaster cDNA clone Cp_aI_022_N17 3', mRNA sequence	0.86	8%	41.67%	*Cynops pyrrhogaster*
P8	FS312050.1	Cp_aORL Cynops pyrrhogaster cDNA clone Cp_aORL_003_G19, mRNA sequence	0.60	27%	27.12%	*Cynops pyrrhogaster*
P9	FS297113.1	Cp_aI Cynops pyrrhogaster cDNA clone Cp_aI_019_L20 3', mRNA sequence	0.76	14%	45.45%	*Cynops pyrrhogaster*

tBLASTn (NCBI) was used to find unannotated nucleotide sequences in the indicated species.

*Gene ID: Corresponds to the symbol used for each predicted zebrafish protein subjected to bioinformatics analysis, the query. Only those with hits are shown.

**Description: Describes the unannotated ortholog found for the gene.

†E Value: The Expect value (E-value) or random background noise is the number of hits one can "expect" to see by chance when searching a database of a particular size (https://blast.ncbi.nlm.nih.gov). The lower the E-value, or the closer it is to zero, the more "significant" the match is. We applied a cut-off of E-value < 1.

‡Query cover: The percentage of the query's sequence (zebrafish gene) that overlaps the subject's sequence (returned ortholog).

§Identity: The percentage of amino acids within the covered part of the query that are identical between the query and the returned ortholog.
